# Multi-omics integration reveals hypoxia-driven mechanisms in vascular dementia: a machine learning and single-cell sequencing approach

**DOI:** 10.1097/MS9.0000000000004296

**Published:** 2025-11-12

**Authors:** Zhibo Xuan, Huasen Yang, Lining Duan, Xian Wu, Mengwan Hu, Weiwei Shan

**Affiliations:** a Graduate School, Heilongjiang University of Chinese Medicine, Harbin, China; b Department of Acupuncture and Moxibustion, First Affiliated Hospital of Guangzhou University of Chinese Medicine, Guangzhou, China; c Department of Acupuncture and Moxibustion, First Affiliated Hospital of Heilongjiang University of Chinese Medicine, Harbin, China; d Department of Gastroenterology, First Affiliated Hospital of Heilongjiang University of Chinese Medicine, Harbin, China

**Keywords:** bioinformatics, hypoxia, machine learning, single-cell sequencing analysis, vascular dementia

## Abstract

**Background:**

This study aimed to identify and analyze hub hypoxia-related genes in vascular dementia (VaD) and explore their roles in metabolism, immune response, and cell differentiation, thereby offering potential biomarkers and therapeutic targets.

**Methods:**

Using VaD datasets (GSE122063, GSE282111) from Gene Expression Omnibus (including high-throughput and single-cell sequencing data), analyses were performed via R preprocessing, WGCNA, and machine learning. A chronic cerebral hypoperfusion model was established by two-vessel occlusion (2VO), with verification through immunohistochemistry.

**Results:**

WGCNA identified 7451 module genes and 36 overlapping hypoxia-related genes; machine learning pinpointed *DUSP1, MAFF*, and *TGFBI* as hub genes. ssGSEA linked these genes to metabolic pathways (e.g., cysteine-methionine metabolism, glycolysis) and cell death pathways (apoptosis, pyroptosis). They associated with immune cells like M2 macrophages and neutrophils. Single-cell analysis showed their expression in astrocytes, endothelial cells, and microglia, with endothelial cells exhibiting a hypoxic phenotype via pathways like PI3K-Akt. Immunohistochemistry revealed increased *DUSP1*, *MAFF* and *TGFBI* in models.

**Conclusions:**

This study identified *DUSP1, MAFF*, and *TGFBI* as key players in hypoxia-related mechanisms in VaD, highlighting their pivotal roles in metabolic regulation, cell death pathways, immune microenvironment modulation, and neural differentiation. These insights enhance our understanding of VaD pathogenesis and suggest that these genes may be potential therapeutic targets for cognitive impairment.

## Introduction

Vascular dementia (VaD) is an acquired syndrome characterized by a progressive decline in cognitive ability resulting from cerebral hypoperfusion and cerebrovascular disease^[1]^. This condition manifests as impairments in learning and memory, as well as motor, language, and personality disorders, severely affecting patients’ quality of life and overall well-being, thereby imposing a substantial burden on families and society. In China, with an increasing trend of population aging, approximately 20.8% of the elderly population over 65 years of age suffers from mild cognitive impairment, with up to 42% of these cases attributable to cerebrovascular disease. VaD is the second most common type of dementia after Alzheimer’s disease, accounting for 15% of all dementia cases^[2]^. Research has shown that VaD pathogenesis is closely associated with oxidative stress, neuroinflammation, and amyloid β-protein (Aβ) deposition^[3]^.

**HIGHLIGHTS**

- Identified *DUSP1, MAFF, TGFBI* as hub hypoxia-related genes in vascular dementia (VaD)
- Integrated multi-omics, machine learning, and single-cell sequencing to explore VaD pathogenesis
- Hub genes linked to metabolism, immune response, and cell death pathways in VaD
- Endothelial cells show prominent hypoxic phenotype in VaD at single-cell resolution

Hypoxia is defined as a condition in which oxygen levels available to the body or a specific region fall below normal physiological requirements. Given the dependence of the human body on oxygen, there is an increased sensitivity to hypoxic conditions. The brain, which is the organ most dependent on oxygen, accounts for 20% of the total oxygen consumption of the body, making it particularly susceptible to the effects of hypoxia. Hypoxia is implicated in various neuropathological processes including Alzheimer’s disease, Parkinson’s disease, and VaD. Research suggests that hypoxia exerts a bidirectional impact on the brain, while prolonged hypoxia can lead to irreversible brain damage. Intermittent hypoxia and hypoxia–hyperoxia can induce physiological adaptations that enhance resistance to subsequent hypoxic and ischemic injuries. This presents potential therapeutic benefits for various pathological conditions^[4]^. Therefore, investigating hub hypoxia-related genes and their potential mechanisms in VaD are important for future therapeutic interventions.

This study conducted a comprehensive bioinformatic analysis of high-throughput and single-cell sequencing datasets for VaD, integrating machine learning to identify hub hypoxia-related genes that were validated through animal experiments. This study aimed to explore the underlying genetic mechanisms involved in cell death, metabolism, immune cell infiltration, and VaD progression. This study reveals potential therapeutic targets for alleviating cognitive dysfunction in patients with VaD.

## Methods

### Data source

Microarray data pertaining to the disease were sourced from the Gene Expression Omnibus database^[5]^. The high-throughput sequencing dataset selected for analysis was the GSE122063 (GPL16699) dataset, comprising 36 disease and 44 normal samples. The single-cell dataset was derived from GSE282111 (GPL24676) and encompassed four normal and four diseased samples.

### Data preprocessing

R programming language was used to preprocess the gene expression microarray dataset, which involved tasks such as annotating gene names, managing missing values, and addressing duplicate values. For processing gene expression data, the maximal mean method was used to resolve duplicate gene names. The “rowMeans()” function was applied to calculate the mean expression value for each record, which was subsequently added as a new column in the dataset. The “group_by()” function from the “dplyr” package was used to group data by gene name (ID), and the “slice_max(order_by = MeanExpression,n = 1)” function was used to select the row with the highest mean expression value. This approach retains the most representative gene expression record, ensuring that only the record with the highest expression value for each gene is preserved in the dataset. This method is particularly suitable for analytical scenarios in which the focus is on the strongest gene expression in the sample.

### Differential expression analysis

Differential expression analysis was performed using the limma package^[6]^ in R software. The criteria for selecting differentially expressed genes (DEGs) were established at *P* < 0.05, with the dynamic log2 fold change (log2FC) threshold defined as |log2FC| > [mean(|log_2_FC|) + 2sd(|log_2_FC|)]^[6]^. These DEGs were subsequently selected for further investigation to identify genes exhibiting altered expression between diseased and normal samples.

### Weighted Gene Co-expression Network Analysis (WGCNA)

WGCNA was conducted on the GSE122063 dataset using the WGCNA package^[7]^ in R to investigate the correlation between the modules and diseases. The top 6000 DEGs with an average expression level of at least 1 were selected by applying an outlier sample value cutoff of 0.98, retaining 98% of the samples for further analysis. The “pickSoftThreshold” function from the WGCNA package was employed to determine the optimal soft threshold, facilitating the division of data into distinct modules. Clustering was used to merge similar modules, with a minimum module size set at minModuleSize = 100 and deepSplit = 2 for the cut-off depth. The module most strongly associated with VaD was identified as the key module for subsequent analyses.

### Gene analysis and enrichment analysis

Hypoxia-related genes were sourced from the HALLMARK gene set within the GSEA database (MSigDB, www.gsea-msigdb.org), comprising 200 genes. To identify hub hypoxia-related genes pertinent to VaD, these genes were cross-referenced with the key modules derived from WGCNA. Enrichment analysis of the intersecting genes was conducted using the clusterProfiler package^[8]^ in R. A significance threshold of *P* < 0.05 was established for screening the results, and a bubble chart was generated to illustrate the significant biological processes (BP), cellular components (CC), molecular functions (MF), and pathways associated with the disease.

### Machine learning screening

In this study, nine machine learning algorithms were employed to identify hub genes based on the expression of DEGs in the dataset. The methods used included Random Forest (RF), Support Vector Machine (SVM), extreme Gradient Boosting (XGB), Generalized Linear Model (GLM), Gradient Boosting Machine (GBM), K-Nearest Neighbors (KNN), Neural Network (NNET), Least Absolute Shrinkage and Selection Operator (LASSO), Decision Tree (DT), and C5.0 Decision Tree algorithm (C50). To assess the efficacy of these models, residual boxplots, cumulative distribution plots of residuals, and Receiver Operating Characteristic (ROC) curves were generated. Two models demonstrating superior performance were used to identify hub genes.

### Hub gene analysis

ssGESA was performed on disease samples and feature genes using metabolism^[9]^ and cell death pattern^[10]^ datasets. The minimal gene set was established at five genes, and the enrichment scores for each gene set in each sample were computed, resulting in an enrichment score matrix. The CIBERSORT analysis technique^[11]^ was employed to assess the level of immune cell infiltration between diseased and normal samples in the GSE122063 dataset. Box plots were used to display the differential expression of each result, and Laplace charts were constructed to illustrate the correlation of feature genes with the analysis results.

### Single-cell analysis

The dataset was processed using the Seurat package^[12]^ in the R programming environment. The CreateSeuratObject() function was employed with parameters min.cells = 3 and min.features = 200 to exclude cells with fewer than 200 genes and retain genes expressed in a minimum of three cells, while filtering out cells exhibiting overexpression of mitochondrial genes (percentage.mt > 20%). The FindVariableFeatures() function was used to identify the top 2000 variable genes. For dimensionality reduction analysis, the RunPCA() function was applied, and the elbow plot () function was used to determine the principal components. The cluster package^[13]^ was used to determine optimal resolution. Six types of brain cells were annotated: astrocytes, endothelial cells, microglia, neurons, oligodendrocyte progenitor cells (OPCs), and oligodendrocytes. Table 1 provides detailed information on the marker genes for all cell types^[14]^. The AUCell package^[15]^ was used to analyze the hypoxic metabolic levels in each cell. The Monocle3 package^[16]^ was used for pseudotime analysis to examine hub gene trends. The velocyto.R package^[17]^ was used for RNA velocity analysis of core genes.

**Table 1:** Markers of major cell types in the human brain

Cell type	Marker
Astrocytes	GFAP, ALDH1L1, SOX9, AQP4, ATP13A4, CBS
Endothelial cells	CLDN5, ADGRL4, ITM2A, ESAM, A2M, APOLD1, SPARC
Microglia	CX3CR1, ADRB2, CCL3, CSF1R, P2RY12, SALL1, ADORA3, CD68
Neurons	SLC17A7, STMN2, SYT1, SYN1, C14orf37, CDO1, CNTN4, COBL, DCN, DLX1, DLX2, DLX5, DLX6, TMEM130
Oligodendrocytes	MAG, MOG, MOBP, OLIG2, UGT8, CNP, FA2H
OPCs	PDGFRA, MMP15, NEU4, FYN, NNAT

### Animals

Eight-week–old male Sprague-Dawley rats weighing (200 ± 20) g were procured from the Safety and Assessment Center of a university of Chinese medicine. All experimental procedures and rat use were approved by the Experimental Animal Welfare Ethics Committee of the Heilongjiang University of Chinese Medicine (Approval No. 2024120901). The rats were housed in an SPF-grade laboratory for a 1-week acclimatization period, with environmental conditions maintained at a temperature range of 25–27 °C and relative humidity between 50% and 70%. This work has been reported in line with the ARRIVE criteria^[18]^.

### Laboratory apparatus

The behavioral apparatus and experimental instruments utilized in this study were obtained from the following commercial sources: The Morris water maze system was provided by Shanghai Xinruan Information Technology Co., Ltd. (Shanghai, China). Additional equipment included a DH-250 electric thermostatic incubator (Beijing Kewei Yongxing Instrument Co., Ltd., Beijing, China), a SpectraMax-190 full-wavelength microplate reader (Molecular Devices LLC, USA), an eBlot™ L1 rapid wet transfer system (GenScript Biotech Co., Ltd., Nanjing, China), and an iCEN-24R high-speed refrigerated centrifuge (Hangzhou Ausheng Instrument Co., Ltd., Zhejiang, China). Microscopic analysis was performed using an ECLIPSE Ni-U upright microscope (Nikon Corporation, Tokyo, Japan). Tissue processing was facilitated by a YD-1508B manual tissue slicer (Yidi Medical Equipment Factory, Jinhua, China) and a KH-LQ3500 cryostat microtome (Kuohai Medical Technology Co., Ltd., Xiaogan, China).

### Experimental design and administration

Five rats were randomly assigned to the sham group, and the other ten were designated as the model group for VaD induction. The model was established through bilateral common carotid artery ligation (2-vessel occlusion, 2VO)^[19]^, thereby creating a chronic cerebral hypoperfusion (CCH) rat model to simulate VaD. Following a 12-hour fasting period, the rats were anesthetized via an intraperitoneal injection of Zoletil 50(R) (30 mg/kg). The central neck hair was removed and disinfected, a 1.5 cm incision was made along the midline of the neck, the subcutaneous tissues were bluntly dissected, and the bilateral common carotid arteries were exposed and carefully separated from the carotid sheath and the vagus nerve. Subsequently, both arteries were double-ligated with a 3-0 silk thread and transected at the midpoint, with continuous monitoring of the rats’ heart rate, respiration, and other vital signs to ensure that no abnormalities occurred before restoring the subcutaneous anatomical structure. The sham group underwent the same surgical procedure without any ligation. At 1 week post-surgery, the Morris water maze experiment^[20]^ was performed for model evaluation using the average escape latency of sham-operated rats as a reference standard. The model was deemed successfully established if the ratio of the average escape latency of the surgical group rats minus the average escape latency of the sham-operated group rats to the average escape latency of the surgical group rats exceeded 20%. Five rats were randomly selected from the successfully modeled rats and divided into the model group. Anesthesia was administered to the rats, and they were subsequently euthanized. The two hemispheres of the brain were promptly separated on ice, and the right hemisphere was preserved in formaldehyde for subsequent analysis.

### Immunohistochemistry

Immunohistochemical staining was used to assess the expression levels of hub genes in the hippocampal tissues of rats from different groups. Paraffin sections were processed with xylene and graded alcohol treatments, followed by a 5-minute rinse in running water and immersion in 3% hydrogen peroxide solution for antigen retrieval. Subsequently, the sections were washed three times with phosphate-buffered saline (PBS) for 5 minutes each, and the primary antibody was applied and incubated overnight at 4 °C. The next day, the cells were rinsed with PBS, and the secondary antibody was added and incubated for 2 hours. After another PBS rinse, DAB was applied and rinsed with PBS. The sections were dehydrated using graded alcohol, sealed with resin glue, observed under a microscope, and photographed with a digital camera. ImageJ software was used to calculate the area.

## Results

### Differential gene analysis results

**Figure 1.**
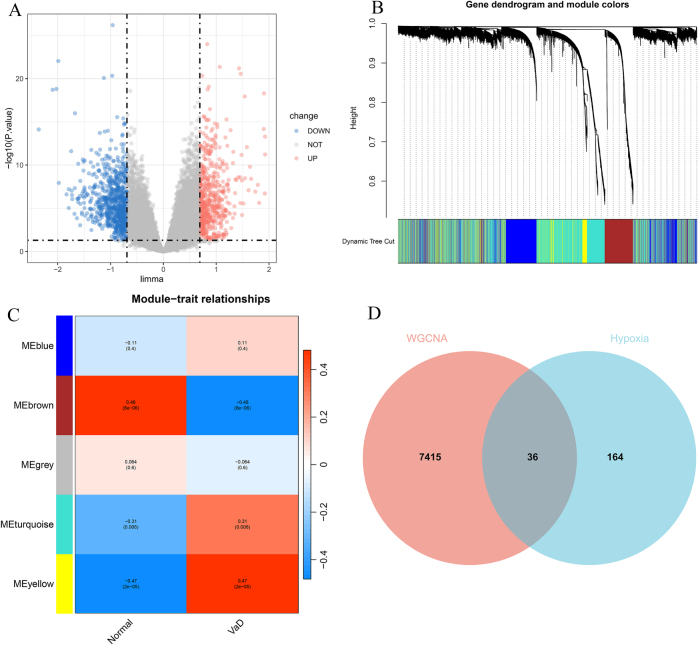
(A) Volcano plot; (B) Gene clustering dendrogram and module visualization; (C) Module-trait association analysis plot; (D) Venn diagram.

In this study, a comprehensive multistep methodology was employed to identify hub genes with high relevance to VaD. The dynamic log2FC threshold for GSE122063 was established at 0.690, resulting in the identification of 1389 DEGs, of which 817 were downregulated and 572 were upregulated. A volcano plot of the DEGs was generated (Fig. 1A). Using WGCNA, multiple gene modules were successfully identified using a dynamic tree-cutting method, with the genes within each module exhibiting functional similarity (Fig. 1B). The brown, turquoise, and yellow modules, which demonstrated significant associations with disease relevance, were selected (Fig. 1C), yielding 7451 module genes. This network analysis facilitates the identification of patterns of gene co-expression, thereby highlighting genes that may be functionally relevant and potentially significant in VaD. Finally, hypoxia-related genes were intersected with WGCNA module-associated genes (Fig. 1D), identifying 36 genes for further analysis.

### Enrichment analysis

**Figure 2.**
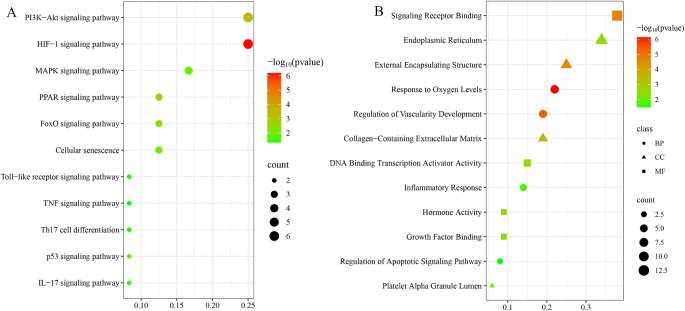
(A) Bubble plot of KEGG; (B) Bubble plot of GO.

Further examination of these 36 hub genes was performed using Kyoto Encyclopedia of Genes and Genomes (KEGG; Fig. 2A) and Gene Ontology (GO) analyses (Fig. 2B). The results of the GO enrichment analysis revealed that these hub genes were predominantly enriched in BP, such as response to oxygen levels, regulation of vascular development, inflammatory response, and regulation of apoptotic signaling pathways, CC, including the endoplasmic reticulum and platelet alpha granule lumen, and MF, such as signal receptor binding and hormone activity. KEGG enrichment analysis indicated that these hub genes were primarily concentrated in pathways such as hypoxia-inducible factor-1 (HIF-1), phosphatidylinositol 3-kinase-protein kinase B (PI3K-Akt), and mitogen-activated protein kinase (MAPK) signaling pathways.

### Machine learning screening

**Figure 3.**
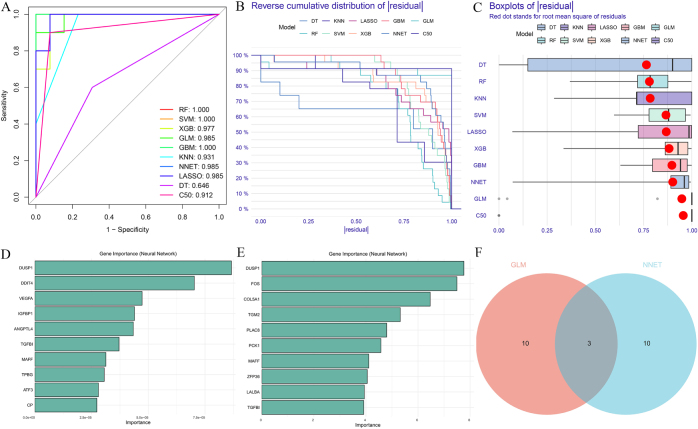
(A) ROC plot, with numbers indicating the area under the curve (AUC); (B) reverse cumulative distribution of residuals; (C) residual box plot, where the box delineates the interquartile range, encompassing the middle 50% of the data from the first quartile (Q1) to the third quartile (Q3). The horizontal line within the box signifies median. The whiskers extend to data points typically beyond 1.5 times the interquartile range (IQR = Q3—Q1). Red dots denote the Root Mean Square of Residuals (RMSE); (D) Results from the Generalized Linear Model (GLM); (E) Results from the Neural Network (NNET); (F) A Venn diagram illustrating the overlap between GLM and NNET results.

Based on the ROC curve analysis results, models with an AUC of 1 that presented an overfitting risk (RF, SVM, and GBM) were excluded from further analysis. Among the remaining models, GLM, NNET, and LASSO exhibited the highest AUC, indicating superior predictive performance for these three models (Fig. 3A). The residual distributions for GLM, KNN, NNET, RF, XGB, and GBM were relatively favorable, with 10% of the residuals exceeding 0.25, 20% exceeding 0.50, and 30% exceeding 0.75, suggesting lower prediction errors for these models (Fig. 3B). Box plots reveal that the median lines in XGB and NNET are relatively centered, and the root mean square is within the box, indicating a concentrated residual distribution. The box lengths for GLM, C50, XGB, and NNET were relatively short, indicating a small range of data fluctuations (Fig. 3C). In summary, the GLM and NNET models were selected for core gene prediction^[21,22]^. Three hub genes were identified: *DUSP1, MAFF*, and *TGFBI* (Fig. 3D-F).”

### Hub gene analysis

**Figure 4.**
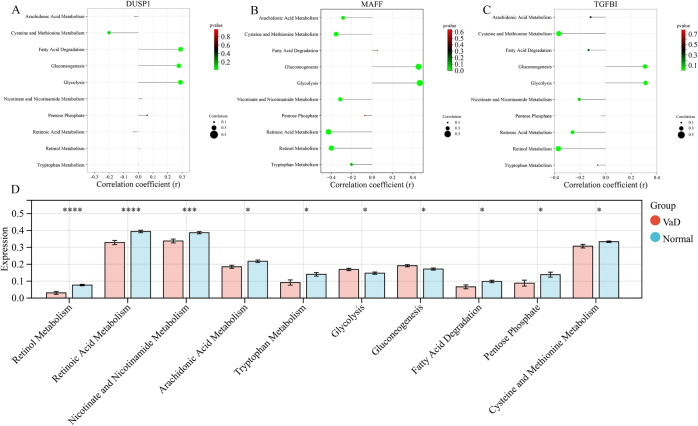
(A) *DUSP1* correlation lollipop chart; (B) *MAFF* correlation lollipop chart; (C) *TGFBI* correlation lollipop chart; (D) Bar chart of metabolism-related entries, where significance is given by *P < 0.05, ***P < 0.001, ****P < 0.0001.

**Figure 5.**
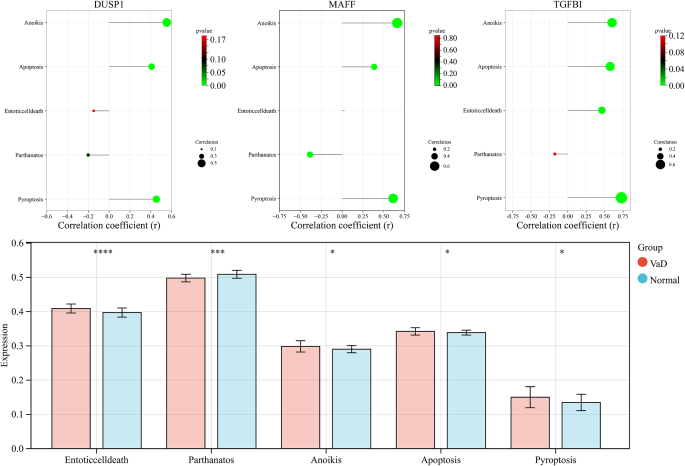
(A) *DUSP1* correlation lollipop chart; (B) *MAFF* correlation lollipop chart; (C) *TGFBI* correlation lollipop chart; (D) Bar chart of cell death entries, where significance is given by *P < 0.05, ***P < 0.001, ****P < 0.0001.

Single-sample Gene Set Enrichment Analysis (ssGSEA) of the metabolic dataset revealed that *DUSP1, MAFF*, and *TGFBI* were negatively correlated with cysteine and methionine metabolism and positively correlated with gluconeogenesis and glycolysis. *MAFF* and *TGFBI* were negatively correlated with retinoic acid and retinol metabolism, whereas *DUSP1* was positively correlated with fatty acid degradation. *MAFF* expression was negatively correlated with arachidonic acid, nicotinate, and nicotinamide metabolism (Fig. 4). Analysis of cell death patterns indicated that *DUSP1, MAFF*, and *TGFBI* were positively correlated with anoikis, apoptosis, and pyroptosis. *TGFBI* was positively correlated with endothelial cell death, whereas *MAFF* was negatively correlated with parthanatosis (Fig. 5). These findings elucidate the intricate roles of *DUSP1, MAFF*, and *TGFBI* in metabolic pathways and cell death patterns, providing significant insights into their potential mechanisms of action in various diseases.

### Immune infiltration analysis

**Figure 6.**
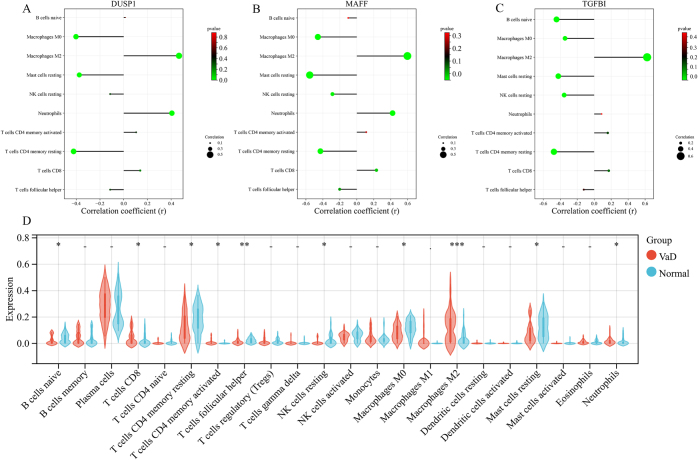
(A) *DUSP1* correlation lollipop chart; (B) *MAFF* correlation lollipop chart; (C) *TGFBI* correlation lollipop chart; (D) Violin plot of immune cells, where *P < 0.05, **P < 0.01, ***P < 0.001.

A correlation analysis was conducted between the selected hub genes and the immune infiltration results of GSE122063. The findings indicated that *DUSP1, MAFF*, and *TGFBI* were negatively correlated with T cells CD4 memory resting, macrophages M0, and mast cell activation and positively correlations with macrophages M2. Additionally, *DUSP1* and *MAFF* were positively correlated with neutrophils, whereas *MAFF* and *TGFBI* were negatively correlated with resting NK cells, with all correlations being significant (*P <* 0.05; Fig. 6A–C). Furthermore, significant differences were observed between the disease and healthy groups in terms of naive B cells, T cells CD4 memory resting, NK cells resting, macrophages M0, macrophages M2, mast cells activated, and neutrophils (Fig. 6D).

### Single-cell analysis results

**Figure 7.**
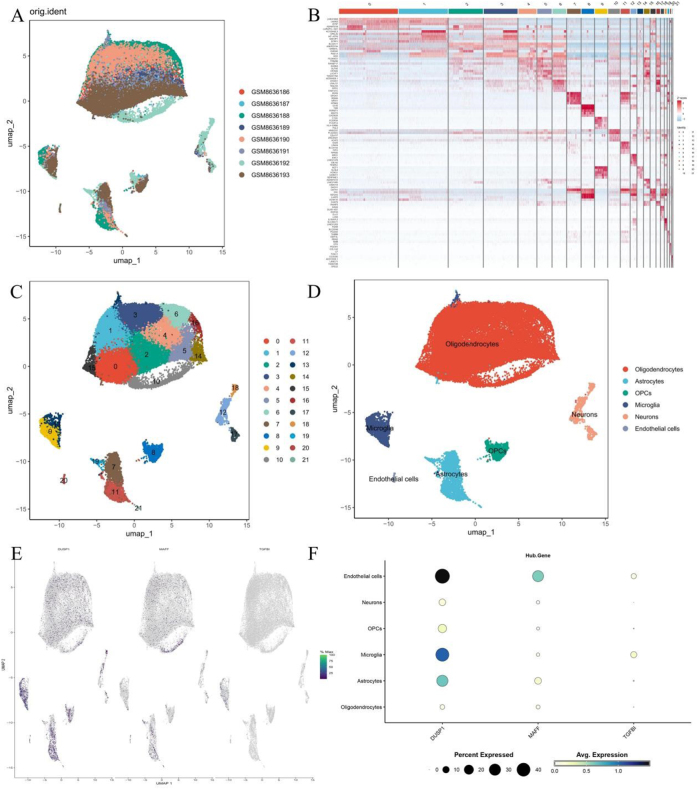
(A) UMAP after clustering (colored by cell cluster); (B) clustering heatmap; (C) UMAP after clustering (colored by group); (D) brain cell annotation map; (E) gene distribution heatmap; (F) gene expression bubble plot.

The GSE282111 dataset comprised 36 353 cells, with 17 177 cells derived from the healthy group and 19 176 cells from the VaD group. Following quality control procedures, the cell count was reduced to 33 577, including 15 994 in the healthy group and 17 583 in the VaD group (Fig. 7A). The annotation resolution was set to 1, resulting in 21 clusters (Fig. 7B and C), and five cell types within the GSE282111 dataset were successfully annotated (Fig. 7D). The gene distribution bubble chart and gene distribution heat map from cell dynamic simulation (CDS) demonstrated that *DUSP1* was expressed in various cells, with significant expression in endothelial cells, astrocytes, and microglia; *MAFF* was predominantly expressed in endothelial cells and astrocytes; whereas *TGFBI* was expressed at low levels in microglia and endothelial cells (Fig. 7E and F).

**Figure 8.**
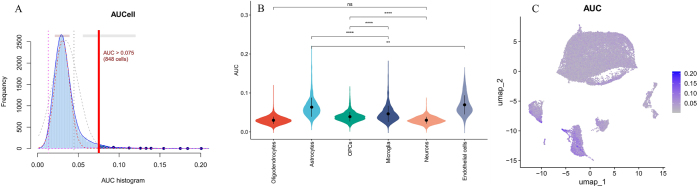
(A) AUCell scoring; (B) AUCell scoring violin plot; (C) AUCell scoring UMAP distribution plot.

In this study, the AUCell algorithm was used to conduct hypoxia-related gene set activity scoring on single-cell transcriptome data, revealing significant heterogeneity in hypoxia phenotypes among different cell types. The AUC score threshold was set at 0.075, with 848 cells exceeding this threshold (Fig. 8A). Notably, endothelial cells exhibited the most significant hypoxic phenotype activation, which was markedly higher than that in neurons and astrocytes (Fig. 8B). This activation may be related to blood-brain barrier (BBB) dysfunction. Previous analyses indicated that the hypoxia-related genes *DUSP1* and *MAFF* were significantly expressed in endothelial cell subpopulations with high AUC scores, suggesting that the hypoxic microenvironment may exacerbate cerebrovascular remodeling by inducing the abnormal proliferation of endothelial cells. This study is the first to reveal the hypoxic phenotype specificity of endothelial cells in VaD at the single-cell resolution, providing new insights for targeting the cerebrovascular microenvironment as an intervention strategy.

**Figure 9.**
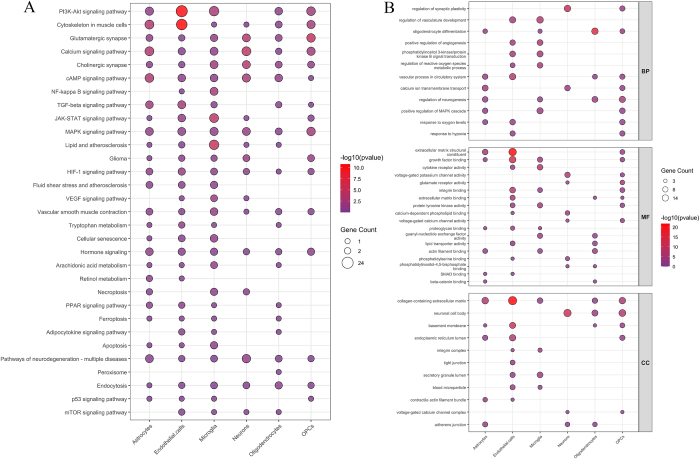
(A) Single-cell KEGG bubble chart. (B) Single-cell GO bubble chart.

We conducted an enrichment analysis of all cell subpopulations. The findings revealed that endothelial cells and OPCs were predominantly enriched in the PI3K-Akt signaling pathway and cytoskeleton in muscle cells, whereas neurons and OPCs were enriched in the MAPK signaling pathway (Fig. 9A). Endothelial cells exhibited enrichment in BP terms such as response to oxygen level, response to hypoxia, and regulation of vascular development. Additionally, they were enriched in CC terms, such as collagen-containing extracellular matrix and endoplasmic reticulum lumen. The results of the MF analysis indicated that endothelial cells, OPCs, and astrocytes were enriched in growth factor binding and extracellular matrix structural constituents, whereas oligodendrocytes and OPCs were enriched in extracellular matrix binding (Fig. 9B). These findings are consistent with those of the aforementioned studies.

**Figure 10.**
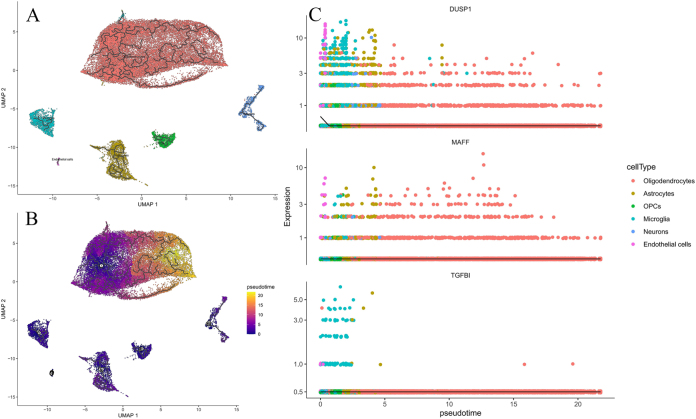
(A) The pseudotime line plot illustrates cell development trajectories; (B) The pseudotime heatmap uses color to represent pseudotime; (C) The gene pseudotime distribution plot features the x-axis as the pseudotime axis and the y-axis as gene expression levels, with lines indicating gene expression trends.

Pseudotime analysis revealed the trajectory of cellular changes (Fig. 10A). The pseudotime heat map depicts the progression of cells from the early to late stages (Fig. 10B). Gene pseudotime analysis demonstrated that the overall expression of hub genes remained stable, with *DUSP1* being relatively highly expressed in endothelial cells, microglia, and astrocytes. *MAFF* exhibited three peaks in astrocytes and endothelial cells, whereas *TGFBI* was relatively highly expressed only in the early pseudotime stages within endothelial cells and microglia, consistent with previous findings (Fig. 10C). These results suggest that the expression of *DUSP1, MAFF*, and *TGFBI* across different cell types is generally stable, with changes exhibiting specific pseudotime and cell-type characteristics, indicating their potential roles in the maturation or differentiation of these cells.

**Figure 11.**
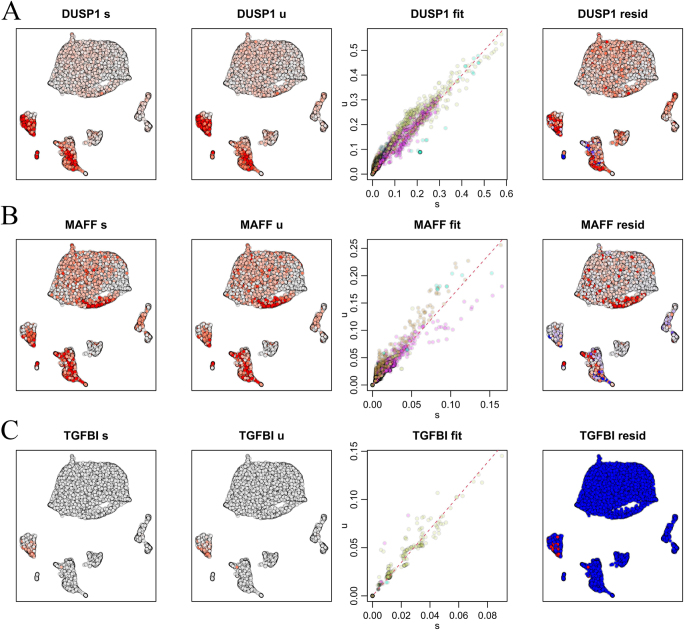
(A), (B), and (C): Results of RNA velocity analysis. u, unspliced mRNA; s, spliced mRNA; fit, fitting plot – the x-axis representing the expression level of spliced mRNA and the y-axis representing the expression level of unspliced mRNA; resid, residual plot, where red cells indicate positive residuals and blue cells indicate negative residuals.

RNA velocity fitting results revealed that spliced and unspliced mRNA of *DUSP1, MAFF*, and *TGFBI* maintained a steady-state relationship in most cells. The residual plots indicated a higher number of red *DUSP1* cells in astrocytes, microglia, neurons, OPCs, and oligodendrocytes, whereas *MAFF* exhibited more red cells in oligodendrocytes. This suggests elevated transcription levels of *DUSP1* and *MAFF* in these cells, with unspliced mRNA levels surpassing the model predictions (Fig. 11A and B). Conversely, *TGFBI* displayed more blue cells across various cell types, suggesting lower transcription levels and potentially indicating transcriptional inhibition or cessation (Fig. 11C). The results demonstrated that the expression changes in *DUSP1, MAFF*, and *TGFBI* were relatively stable, with the fitted curves effectively accounting for the data points, indicating consistent expression changes in these genes across cell populations. These findings provide significant insights into the roles of these genes in cell differentiation and development.

### Immunohistochemistry results

**Figure 12.**
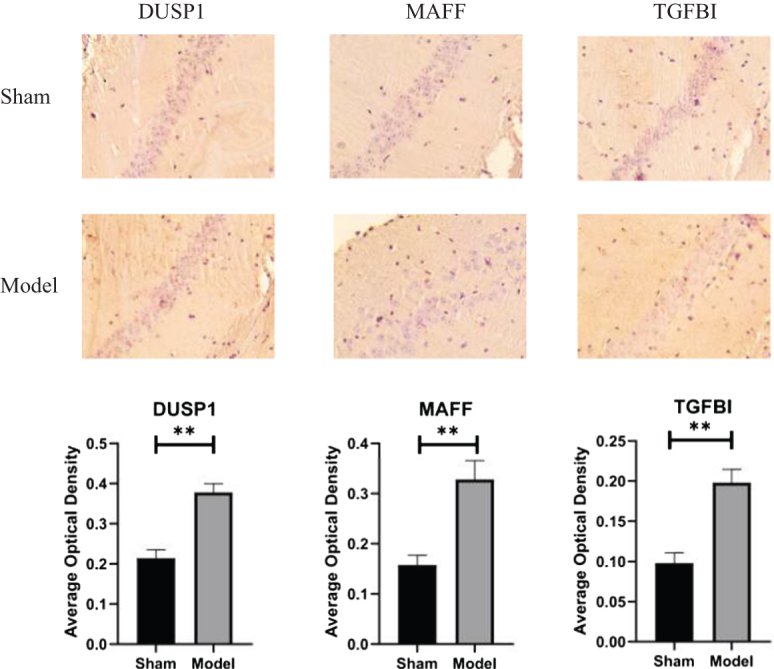
Hub gene immunohistochemistry results (X200). Significance is given by **P < 0.01.

The expression of *DUSP1, MAFF*, and *TGFBI* was observed in the hippocampal tissue of VaD rats using IHC staining. Compared with the sham group, the positive areas for *DUSP1*, *MAFF* and *TGFBI* were significantly increased in the model group (Fig. 12).

## Discussion

VaD is a form of dementia resulting from cerebrovascular diseases, such as stroke and reduced cerebral perfusion, which leads to ischemia of the brain tissue and a decline in cognitive function. The principal pathological alterations include heightened neuroinflammation, oxidative stress, mitochondrial dysfunction, lipid metabolism disorders, and downregulation of growth factor expression^[23]^. Hypoxia is a critical factor in this pathway. Severe or chronic hypoxia exacerbates pathological processes, such as Aβ deposition, tau phosphorylation, and α-synuclein aggregation, by inducing oxidative stress, mitochondrial dysfunction, and neuroinflammation. In contrast, moderate intermittent hypoxic training enhances antioxidant, angiogenic, and neuroprotective effects by activating the HIF and Nrf2 pathways^[4]^. Additional studies have demonstrated that hypoxia leads to a reduction in mitochondrial membrane potential, impairs mitochondrial aerobic respiratory function, and increases intracellular reactive oxygen levels. Hypoxic conditions activate HIF-1α, inducing the expression of pro-inflammatory cytokines such as TNF-α, IL-1β, and IL-6, thereby causing neuroinflammation^[24]^. Consequently, the analysis of key hypoxia-related genes in VaD is essential for targeted prevention and treatment of the disease. Preventive strategies for VaD primarily involve lifestyle interventions, including diet, exercise, cognitive training, and vascular risk monitoring, to improve or maintain cognitive function in older adults^[25]^. Management of hypertension, atherosclerosis, and antiplatelet therapy is crucial for preventing VaD^[26]^. Integrating these measures with targeted treatments based on transcriptomic analyses can provide more precise interventions for patients with VaD.

In this study, a comprehensive bioinformatic analysis of the GSE122063 dataset was conducted. Differential gene expression analysis identified 1389 DEGs, of which 817 were downregulated and 572 were upregulated. WGCNA identified 7451 module genes closely associated with VaD, and volcano plots illustrated the expression patterns of these genes in both diseased and normal samples. Intersection analysis of these genes with hypoxia-related genes identified 36 genes that may play pivotal roles in hypoxia in VaD. GO and KEGG enrichment analyses of these genes revealed their involvement in BP, notably in the regulation of vascular development, inflammatory response, apoptosis signaling pathway regulation, and pathways such as the HIF-1, PI3K-Akt, and MAPK signaling pathways. These findings underscore the significance of the inflammatory response, immune response, cell survival, and cell death, providing deeper insights into the role of hypoxia-related genes in VaD and confirming the established mechanisms of cell death and inflammation.

We used nine machine learning techniques to objectively and comprehensively refine the identification of hub genes, ultimately identifying three key hypoxia-related genes associated with VaD: *DUSP1, MAFF*, and *TGFBI*. Dual-specificity protein phosphatase 1 (*DUSP1*), also referred to as MAPK phosphatase-1 (MKP-1), is located on human chromosome 5q35.1 and encodes a protein with dual-specific phosphatase activity. This protein regulates meiotic cell cycle activity by phosphorylating MAPK MAPK1/ERK2, which plays a pivotal role in the MAPK signaling pathway^[27]^. Previous studies have indicated that *DUSP1* is involved in the regulation of various physiological and pathological processes, including inflammation, immune response, oxidative stress, apoptosis, and metabolism^[28]^. Its expression is induced by hypoxia, inflammatory factors, and oxidative stress, all of which are crucial for maintaining the intracellular homeostasis. Although direct evidence linking *DUSP1* to VaD is lacking, it may contribute to the development of VaD through mechanisms such as the upregulation of *DUSP1* expression by hypoxia-induced HIF-1α activation, inhibition of MAPK family (p38, JNK, and ERK) expression, reduction of mitochondria-dependent apoptosis, and alleviation of neuronal apoptosis and oxidative stress^[29]^. *DUSP1* can negatively regulate the JNK/p38-MAPK pathway, inhibiting the transformation of microglia and macrophages into pro-inflammatory phenotypes, thereby reducing the release of inflammatory factors, such as IL-1β and TNF-α, and mitigating the damage of neuroinflammation to the BBB and neurons^[30,31]^. Chronic cerebral hypoperfusion, a major cause of VaD, maintains the brain tissue in an ischemic hypoxic condition over extended periods, leading to ROS accumulation, impaired axonal plasticity, and exacerbated neuronal damage through MAPK pathway activation. It has been demonstrated that *DUSP1* reduces NADPH oxidase activity, lowers oxidative stress levels, protects mitochondrial function^[17]^, and plays a crucial role in supporting axonal plasticity and survival^[32]^. Furthermore, *DUSP1* can promote endothelial cell survival and angiogenesis through ERK pathway regulation, thereby improving cerebral perfusion following chronic ischemia^[31]^.

The transcription factor MAF bZIP transcription factor F (*MAFF*) is a basic leucine zipper (bZIP) transcription factor located on chromosome 22q13.1, which is part of the MAF family. *MAFF* is regulated by HIF-1α, and under hypoxic conditions, HIF-1α stabilizes and binds to the hypoxia-responsive element in the *MAFF* promoter, thereby activating *MAFF* transcription^[33]^. Atherosclerosis, a significant risk factor for VaD, leads to chronic cerebral hypoperfusion and local ischemia in the brain tissue, forming the pathological basis of VaD. Research has indicated that *MAFF* induces low-density lipoprotein receptor (LDLR) expression under non-inflammatory conditions, promoting cholesterol metabolism and lowering plasma LDL levels. In the presence of inflammatory stimuli, such as lipopolysaccharides, *MAFF* forms a heterodimer with BACH1 and binds to the Maf receptor on the LDLR promoter, resulting in LDLR downregulation^[34]^. *MAFF*’s dual role of *MAFF* under varying conditions suggests its potential in regulating the balance between inflammation and lipid metabolism, which is critically involved in the development and progression of atherosclerosis and cardiovascular disease. Additionally, *MAFF* may activate the IL11 and STAT3 signaling pathways, contributing to angiogenesis and neuroprotective mechanisms, thereby playing a role in VaD^[33]^.

Transforming growth factor-beta (*TGFBI*), also referred to as βig-H3, is an extracellular matrix protein regulated by TGF-β that plays a significant role in various BP, including angiogenesis, migration, invasion, inflammation, lipid metabolism, cell adhesion, and proliferation^[35]^. Research indicates that *TGFBI* expression is markedly upregulated in hypoxic microenvironments through HIF-2α-dependent pathways, which inhibit apoptosis and enhance DNA damage repair by activating the PI3K/Akt signaling pathway^[36]^. *TGFBI* facilitates angiogenesis by activating the NF-κB signaling pathway and is intricately associated with remodeling of inflammatory microenvironments^[37]^. Additionally, *TGFBI* can dynamically regulate plasma concentrations via Stab1/2-mediated clearance mechanisms, thereby inhibiting the activation of monocytes/macrophages, interfering with the migration of inflammatory cells, and exerting anti-atherosclerotic effects^[38]^. Furthermore, *TGFBI*’s role of *TGFBI* in promoting angiogenesis involves the synergistic activation of the spliceosome and lysosome-associated pathways, inhibiting platelet activation, axonal guidance, and cellular stress responses^[39]^. Within the tumor microenvironment, *TGFBI* facilitates tumor angiogenesis and metastasis by remodeling the extracellular matrix and regulating immune cell infiltration. Its hypoxia-dependent expression characteristics suggest that targeting *TGFBI* may be a potential strategy to enhance tumor treatment resistance, although its mechanisms in VaD require further investigation^[36]^.

ssGSEA analysis demonstrated a robust correlation between hub genes and various cell metabolism pathways, including cysteine and methionine metabolism, gluconeogenesis, glycolysis, retinoic acid metabolism, retinol metabolism, fatty acid degradation, arachidonic acid metabolism, nicotinate and nicotinamide metabolism, and cell death pathways, such as anoikis, apoptosis, pyroptosis, and parthanatos. Immune infiltration analysis revealed a significant association between hub genes and diverse immune cell types, highlighting a close relationship between resting memory T cells, CD4 memory resting, Macrophages M0, Mast cells activated, Macrophages M2, neutrophils, and NK cells. These findings underscore the critical roles of cell death, cell metabolism, and immune regulation in VaD, consistent with previous studies. From a cellular metabolism perspective, elevated homocysteine levels promote atherosclerosis and microvascular lesions through oxidative stress, inflammatory responses, and vascular endothelial damage, leading to ischemic hypoxia in the brain tissue, thereby accelerating cognitive dysfunction and increasing the risk of neurodegenerative diseases^[40]^. Short-chain fatty acids enhance BBB integrity and neurotrophin expression by modulating the gut-brain axis, inhibiting NF-κB pathways and oxidative stress, and activating the PI3K/Akt pathways to reduce neuronal apoptosis^[41]^. Long-chain fatty acids inhibit Aβ deposition and abnormal phosphorylation of tau protein, reduce neuroinflammation, and enhance synaptic plasticity and neurocell membrane stability, thereby delaying neurodegeneration through antioxidant, anti-inflammatory, and immune response regulation^[42]^. Arachidonic acid is essential for membrane fluidity, selective permeability, and flexibility; its metabolites mediate inflammatory responses, regulate vascular reactivity, and are closely associated with neurodegenerative diseases^[43]^. Undifferentiated macrophages M0 can polarize into pro-inflammatory M1 or anti-inflammatory macrophages M2. Macrophages M2 are believed to exert anti-atherosclerotic effects by producing anti-inflammatory cytokines and promoting tissue repair^[44]^. Macrophages M2 can perform phagocytosis, clear damaged cellular debris, and secrete neurotrophic factors and anti-inflammatory mediators, including IL-10, TGF-β, and brain-derived neurotrophic factor. Consequently, they have dual roles in anti-inflammatory and neuroprotective processes^[45]^. NK cells, characterized as large granular lymphocytes, are essential for both innate and adaptive immunity and play pivotal roles in immunotherapy^[46]^. Neutrophils interact with platelets to form immunothrombosis, leading to microvascular occlusion and cerebral hypoperfusion, exacerbating BBB damage and neuroinflammation, and further promoting cognitive function decline^[47]^. From an immunological perspective, evaluating immune cell infiltration and determining their composition and differentiation are critical for elucidating the molecular mechanisms of VaD and for exploring novel immunotherapeutic targets. In this study, *DUSP1, MAFF*, and *TGFBI* demonstrated strong positive correlations with Macrophages M2; *DUSP1* and *MAFF* exhibited strong positive correlations with neutrophils, whereas *MAFF* and *TGFBI* showed strong negative correlations with resting NK cells. This suggests that immune cell infiltration occurs in brain tissue during VaD development and that the expression levels of *DUSP1, MAFF*, and *TGFBI* are closely linked to the extent of immune cell infiltration. Single-cell analysis further clarified the distribution of hub genes in central nervous cells, pseudotime states, and mRNA transcription, providing additional evidence for these findings. Bioinformatics analysis revealed that hypoxia-related genes, such as *DUSP1, MAFF*, and *TGFBI*, play pivotal roles in regulating immune cells, cell metabolism, and cell death in patients with VaD, thereby reshaping their immune microenvironment. Although these associations suggest the potential roles of these genes and their functions in VaD, further investigation is warranted, as comprehensive studies have yet to be conducted.

In conclusion, this study integrated bioinformatics, machine learning, and single-cell analyses to identify dual specificity phosphatase 1, transforming growth factor-beta-induced protein, and *MAFF* as pivotal genes regulating hypoxia-related VaD. These genes play essential roles in metabolic regulation, cell death, immune microenvironment remodeling, and differentiation of neural cells. These findings enhance our understanding of the pathological mechanisms underlying VaD hypoxia and suggest that targeting these genes may represent a novel therapeutic strategy for mitigating cognitive dysfunction. Furthermore, the expression profiles of *DUSP1, MAFF*, and *TGFBI* may serve as biomarkers for the early diagnosis and immune microenvironment monitoring of VaD, facilitating precise clinical interventions. However, the study’s limitations include the predominance of samples from Western populations in public databases, which may affect the generalizability of the results because of genetic and lifestyle differences. This necessitates further validation using multiregional and multi-ethnic data. Additionally, the specific mechanisms of hub genes require further investigation using animal models or cell experiments to elucidate the molecular pathways. Future studies should explore the regulatory role of the *DUSP1*-MAPK axis in neuronal survival, the impact of the *MAFF*-mediated lipid metabolism-inflammation interaction network on cerebrovascular hypoxia mechanisms, and the potential value of *TGFBI* in hypoxia-related neural repair in patients with VaD. These investigations provide a theoretical basis for the development of targeted drugs for the treatment of VaD.

## Conclusions

*DUSP1, MAFF*, and *TGFBI* are likely to play significant roles in hypoxia-related pathogenic mechanisms of VaD, thereby offering novel perspectives and potential therapeutic targets for future research. Further investigations are required to elucidate the specific functions of these genes and validate our findings through experiments involving human samples. This study provides valuable insights into the pathological mechanisms associated with VaD and hypoxia and underscores potential new therapeutic targets. By advancing our understanding of these mechanisms, we anticipate the discovery of novel therapies that can effectively manage these challenging conditions.

## Data Availability

Data is available at NCBI GEO: GSE122063 and GSE282111.
